# Supplementary material for: Rationally engineered active sites for efficient and durable hydrogen generation
Source: Nat Commun. 2019 May 23;10:2281. doi: 10.1038/s41467-019-10230-z (PMC6533258; doi:10.1038/s41467-019-10230-z)
Supplement: Supplementary file 1 — Supplementary Information [file 41467_2019_10230_MOESM1_ESM.pdf]

Supplementary Information

Rationally engineered active sites for efficient and durable hydrogen generation

Xue et al.

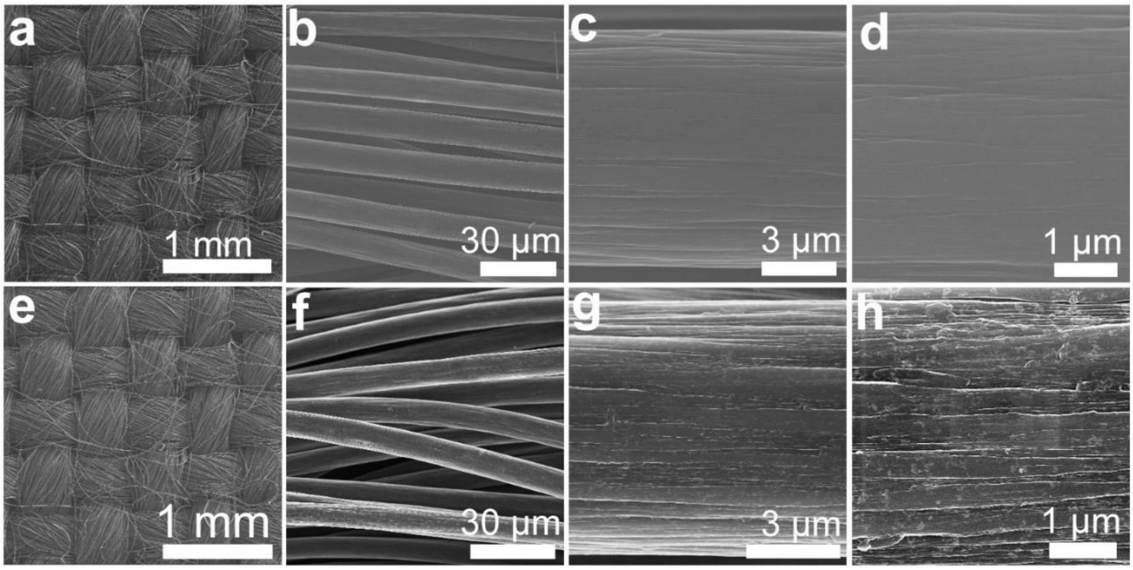

Supplementary Fig. 1 Low and high-magnification SEM images of (a-d) c-CFC and (e-h) A-CFC.

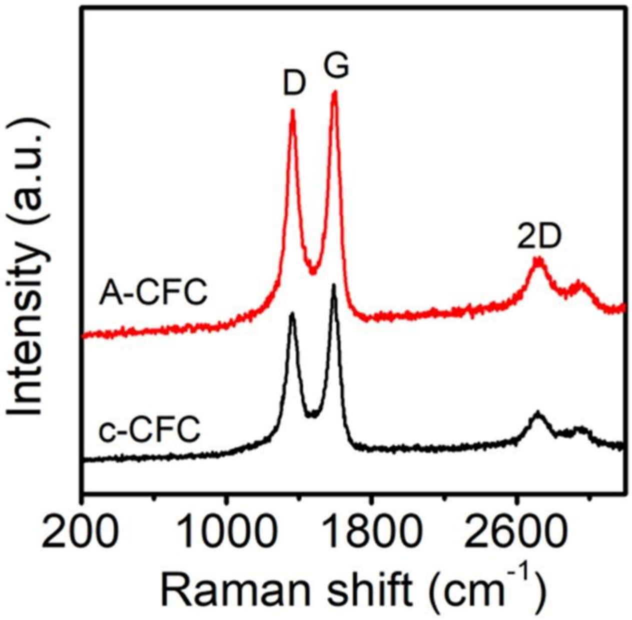

Supplementary Fig. 2 Raman spectra of c-CFC (black line) and A-CFC (red line).

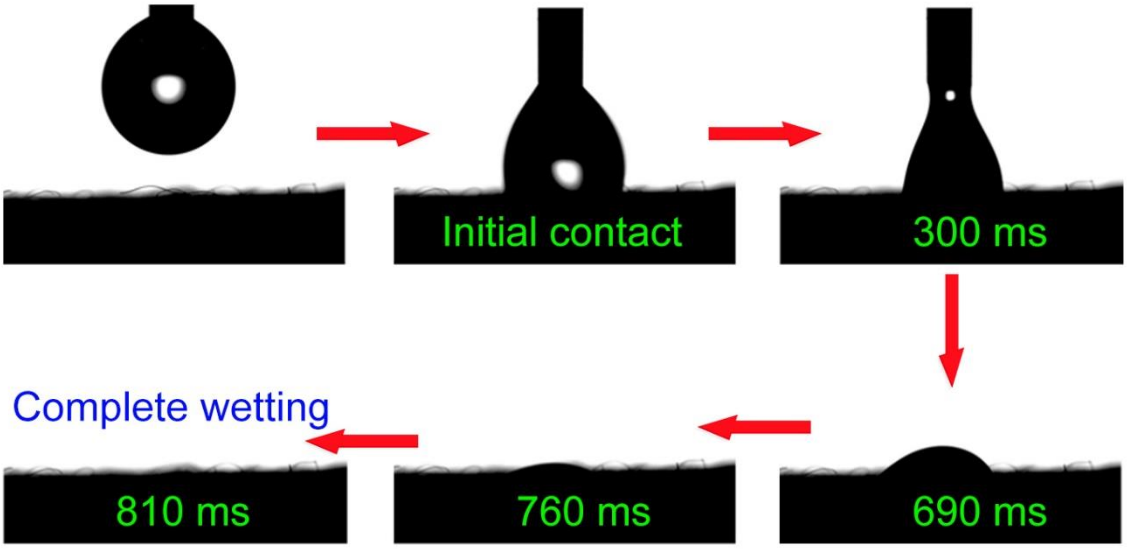

Supplementary Fig. 3 Contact angle measurement on A-CFC surface.

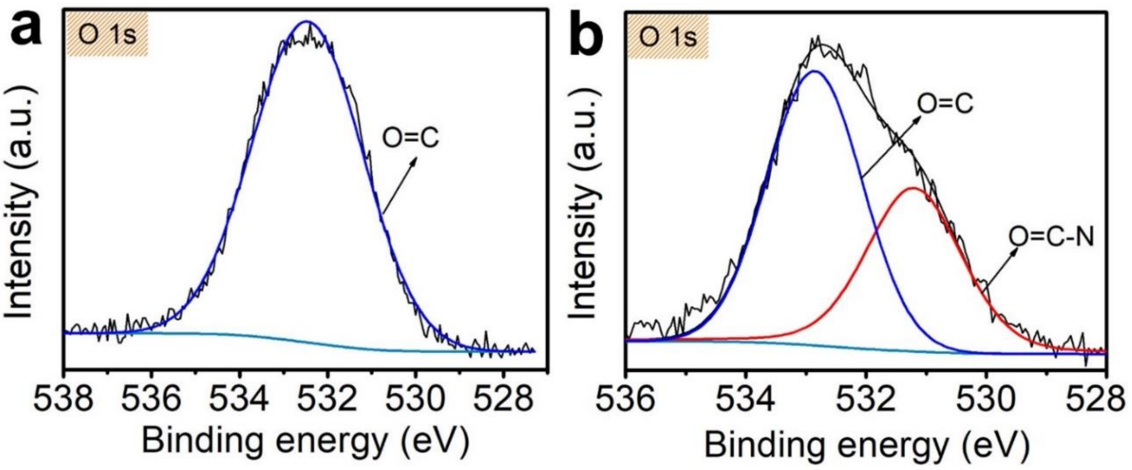

Supplementary Fig. 4 High resolution O 1s XPS spectra of (a) c-CFC and (b) A-CFC, respectively. Compared with pristine c-CFC, a new peak corresponding to the O=C-N group was observed.

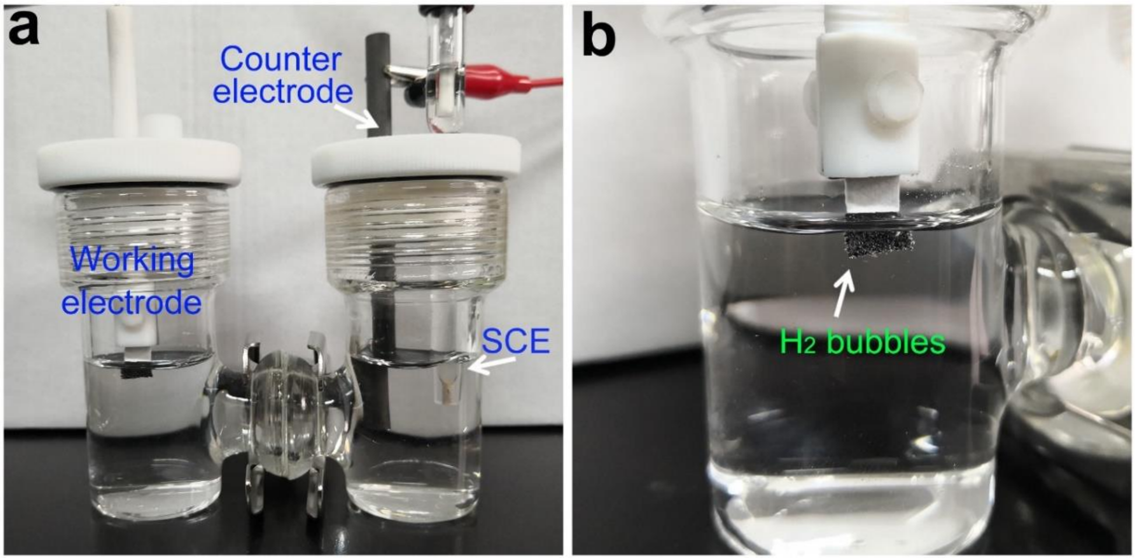

Supplementary Fig. 5 Photographs of (a) the three-electrode cell setup and (b) H_2-evolution during the LSV test.

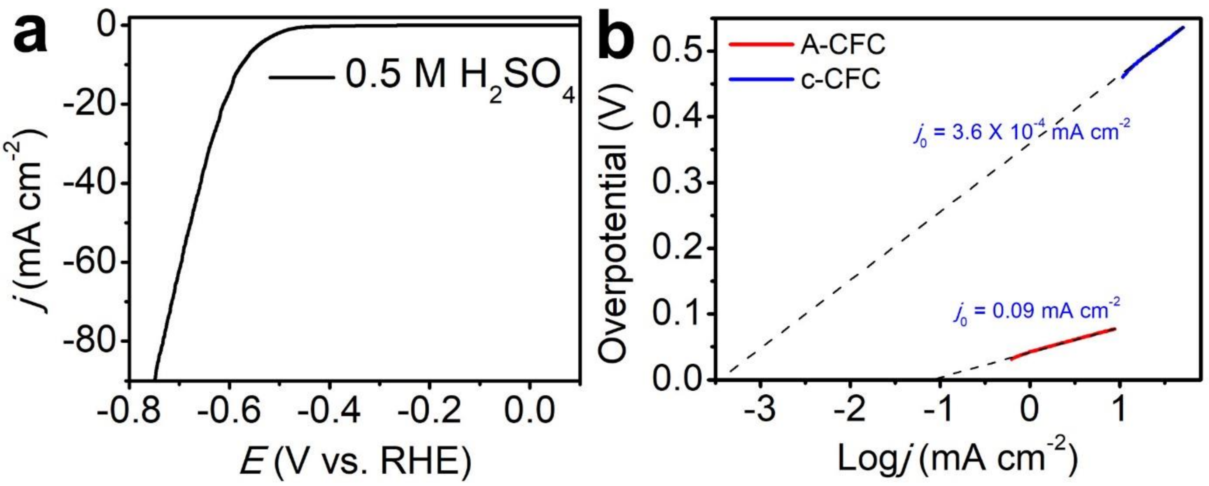

Supplementary Fig. 6 (a) The polarization curves of the catalysts obtained in 0.5 M H_2SO_4. (b) Exchange current density of A-CFC (red line) and c-CFC (blue line) obtained in 0.5 M H_2SO_4.

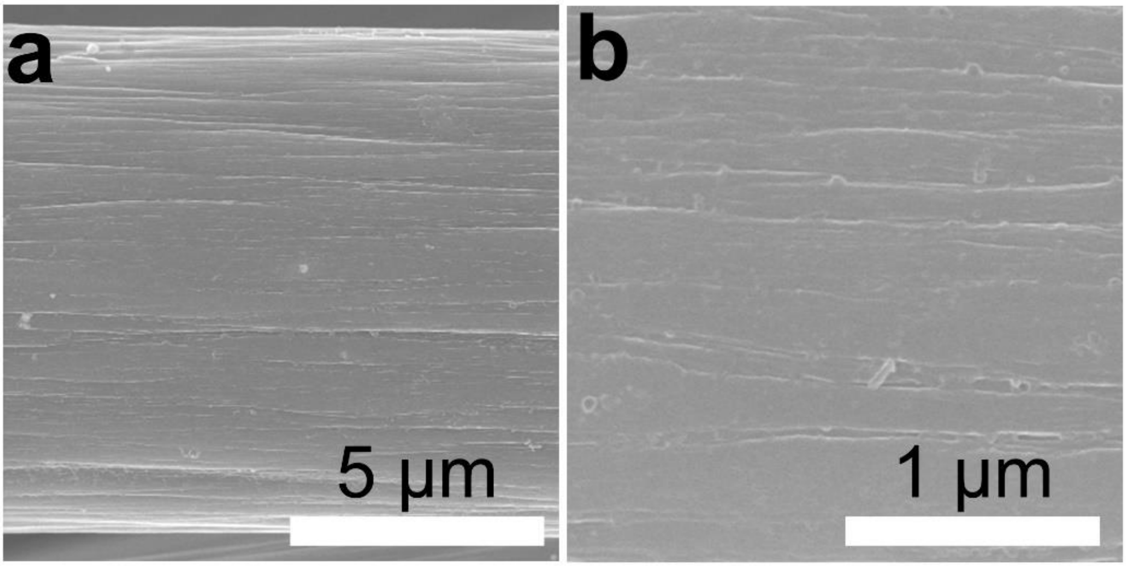

Supplementary Fig. 7 (a,b) SEM images of A-CFC recorded after 13000 cycles in 0.5 M H_2SO_4.

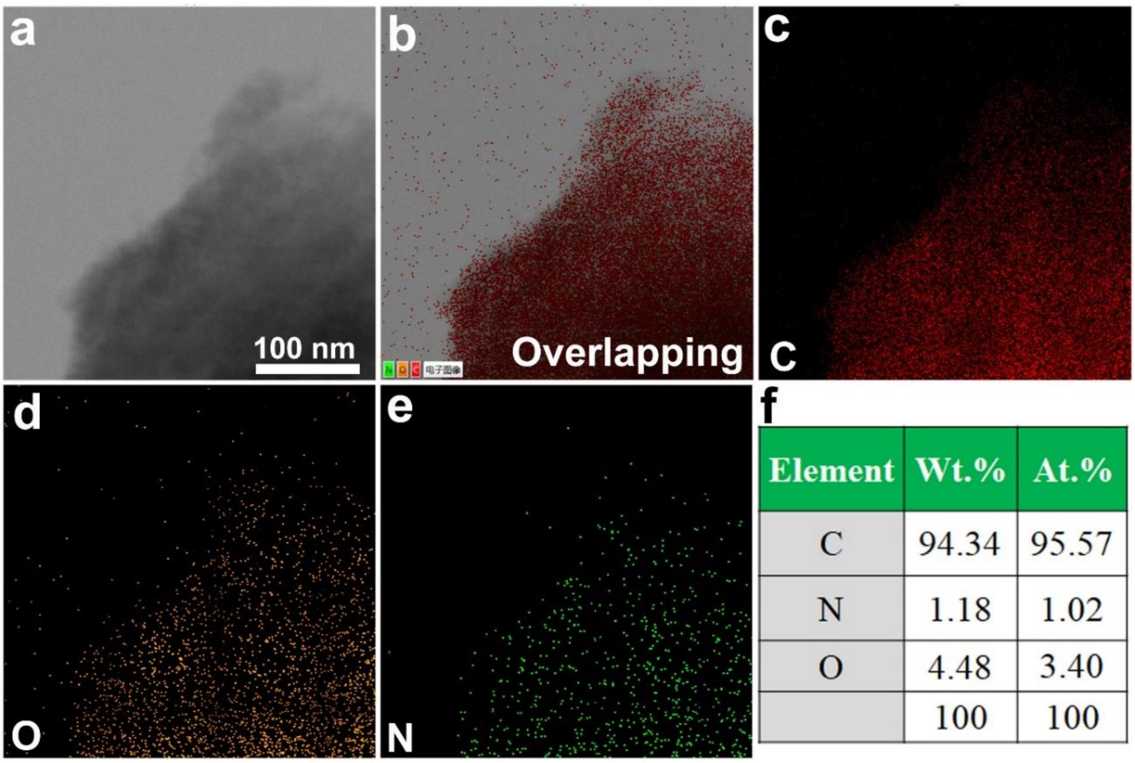

Element	Wt.%	At.%
C	94.34	95.57
N	1.18	1.02
O	4.48	3.40
	100	100

Supplementary Fig. 8 a, Scanning TEM image, **b-e**, elemental mapping, and **f** the percentage of elemental composition in A-CFC obtained after HER test in 0.5 M H_2SO_4.

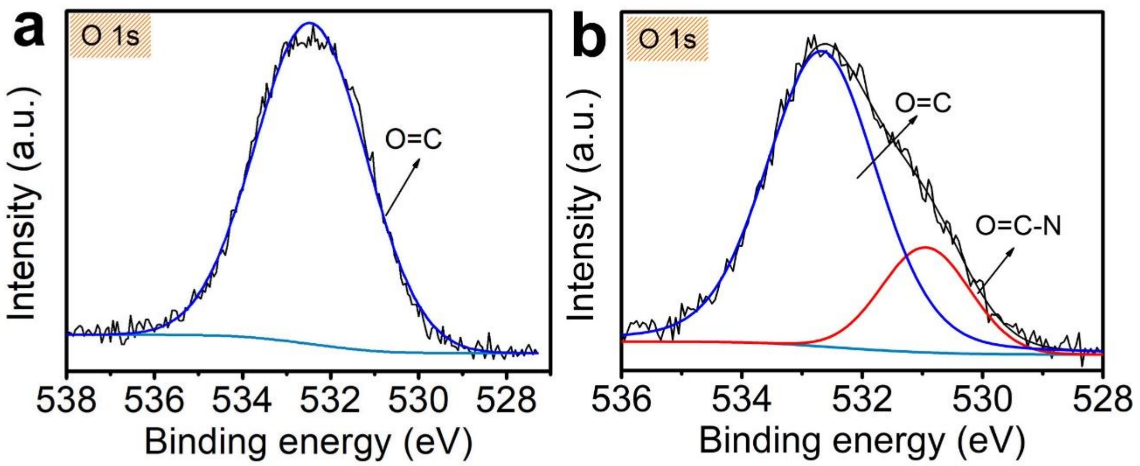

Supplementary Fig. 9 High resolution (a) N 1s and (b) O 1s XPS spectra of A-CFC after 13000 cycles in 0.5 M H_2SO_4.

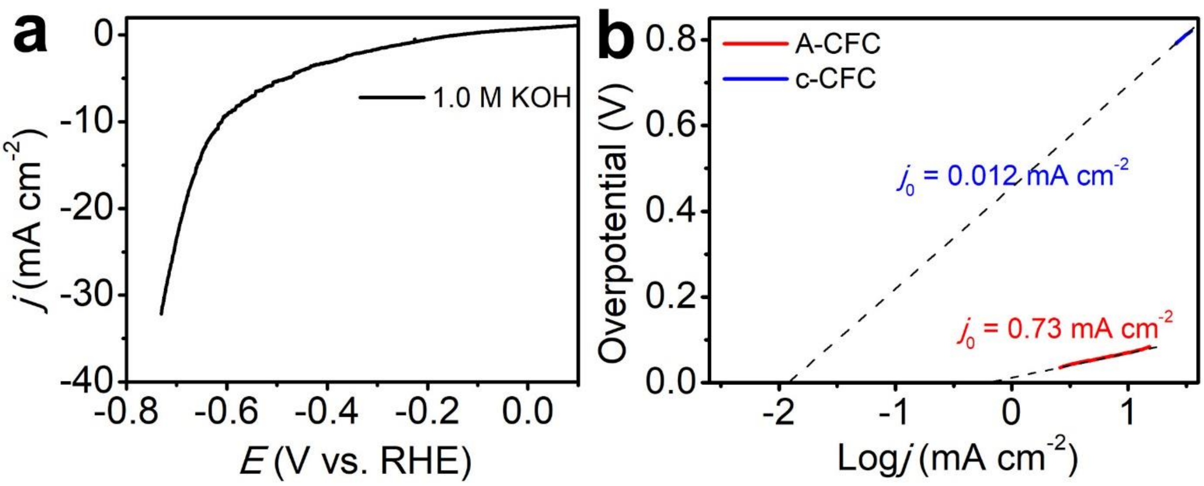

Supplementary Fig. 10 (a) The polarization curves of pristine CFC obtained in 1.0 M KOH. (b) Exchange current density of A-CFC (red line) and c-CFC (blue line) obtained in 1.0 M KOH.

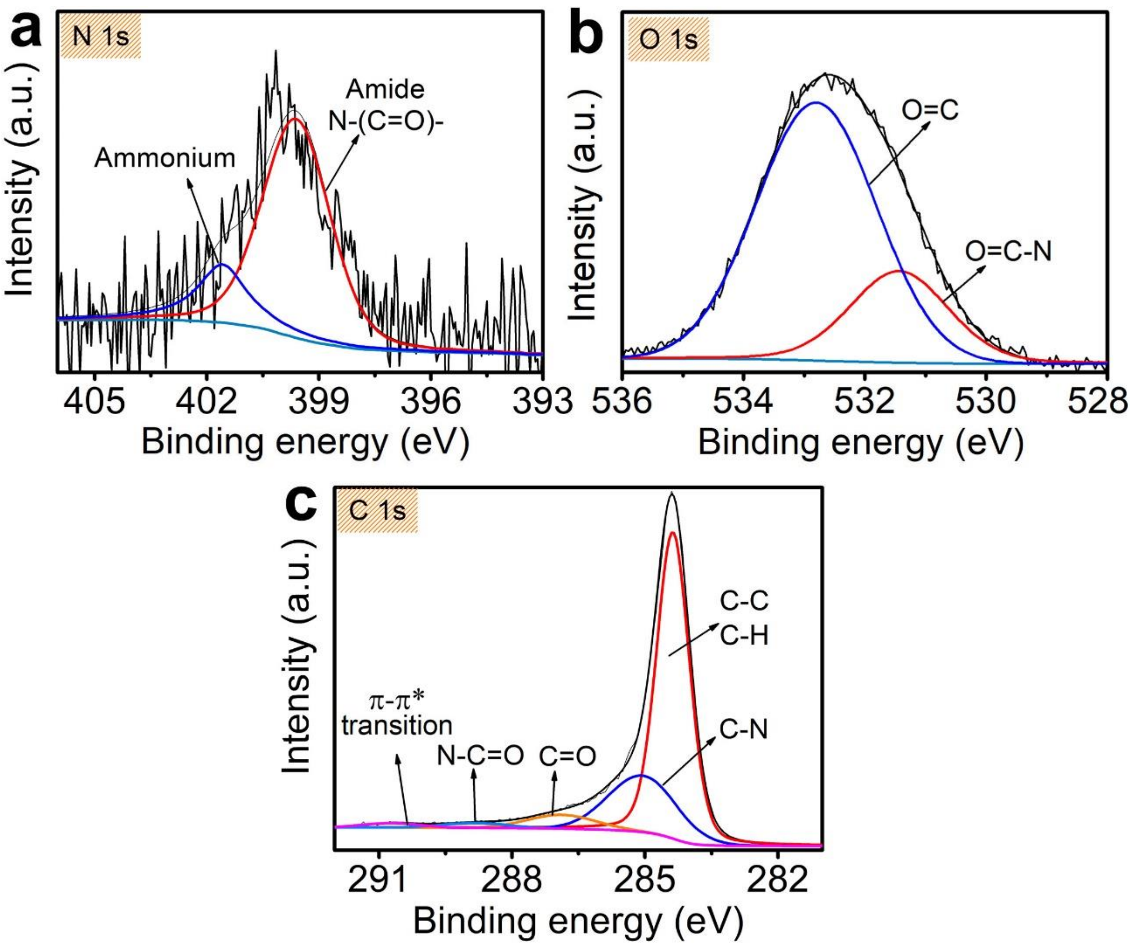

Supplementary Fig. 11 High resolution (a) N 1s, (b) O 1s and (c) C 1s XPS spectra of A-CFC after 18000 cycles in 1.0 M KOH.

The high-resolution N 1s spectrum of the cycled sample showed two peaks at 399.5 and 401.7 eV corresponding to neutral amine nitrogen and ammonium ions, confirming that the nitrogen is still in the amide form even after cycling tests. This was also verified by the O 1s spectra. C1s peak can also be fitted into five peaks at 284.4, 285.2, 286.9, 288.8 and 290.9 eV, respectively, corresponding to aliphatic carbon chains (C-C,H), amine groups (C-N), carbonyl (O=C), carboxyl carbon (O=C-N), and the π-π* transitions in amides. These observations revealed structural stability of the sample.

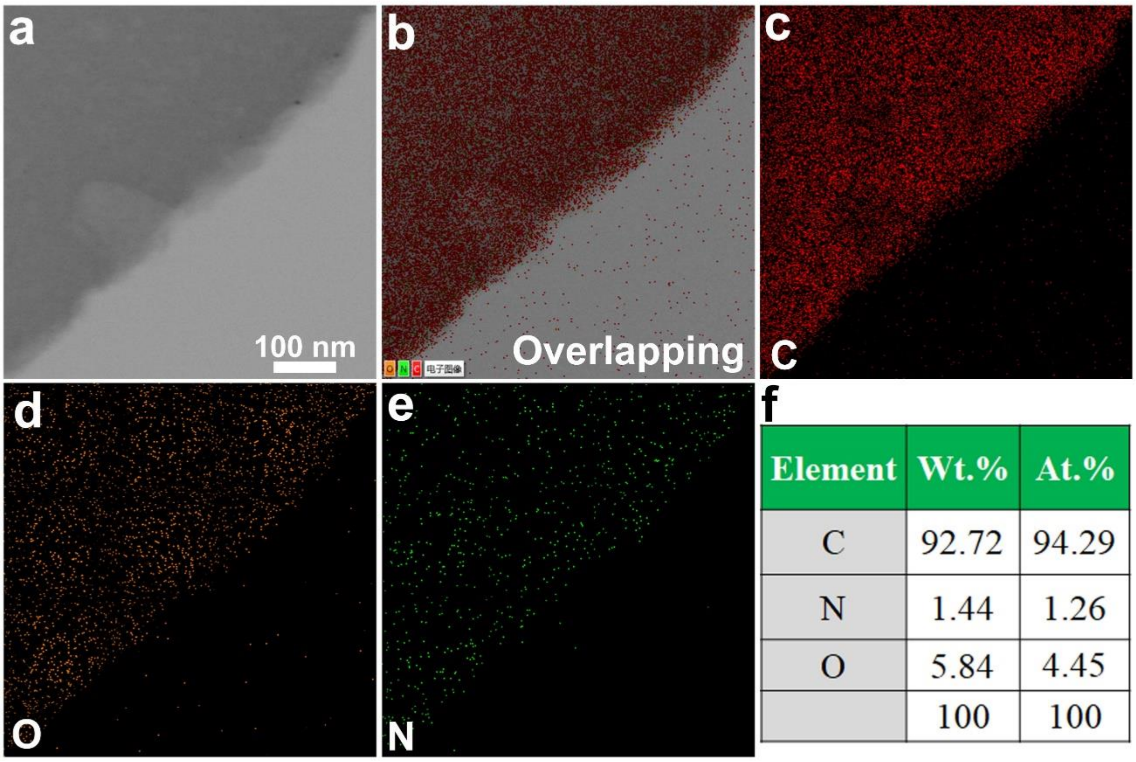

Element	Wt.%	At.%
C	92.72	94.29
N	1.44	1.26
O	5.84	4.45
	100	100

Supplementary Fig. 12 a, Scanning TEM image, **b-e**, elemental mapping, and **f** the percentage of elemental composition in A-CFC obtained after HER test in 1 M KOH.

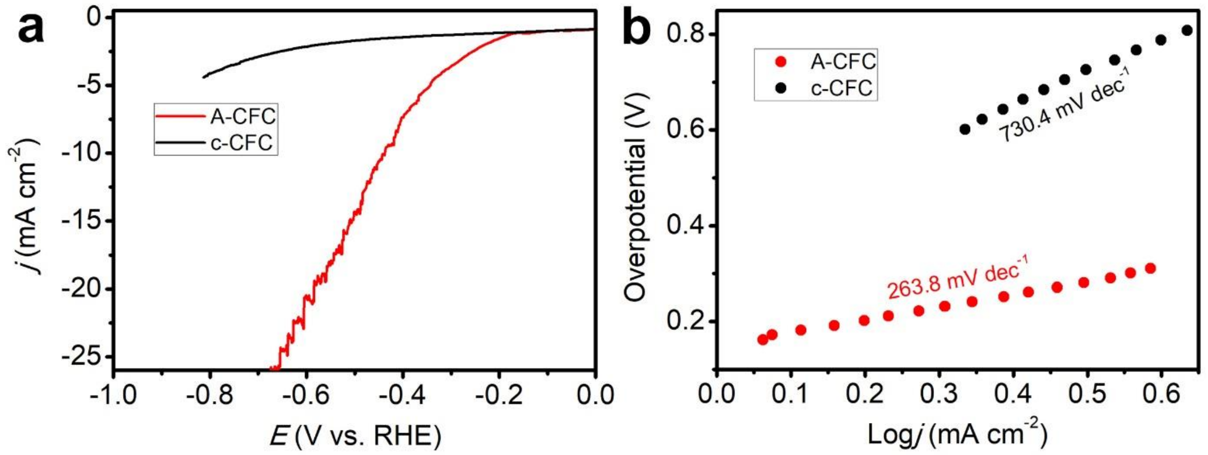

Supplementary Fig. 13 a HER polarization curves and **b** corresponding Tafel plots of catalysts obtained in 1 M PBS.

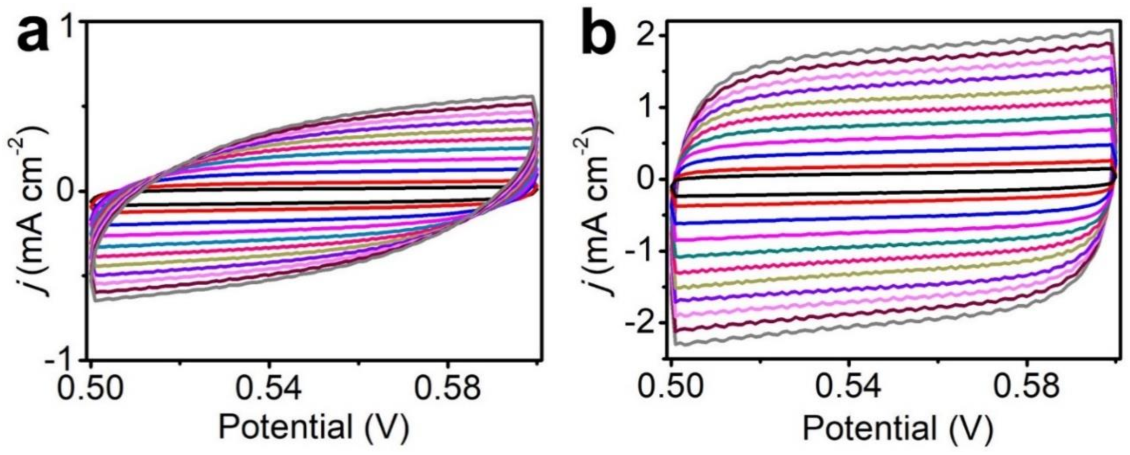

Supplementary Fig. 14 Cyclic voltammetry curves of (a) c-CFC and (b) A-CFC at different scan rates: (—) 10, (—) 20, (—) 40, (—) 60, (—) 80, (—) 100, (—) 120, (—) 140, (—) 160, (—) 180 and (—) 200 mV s^{-1}.

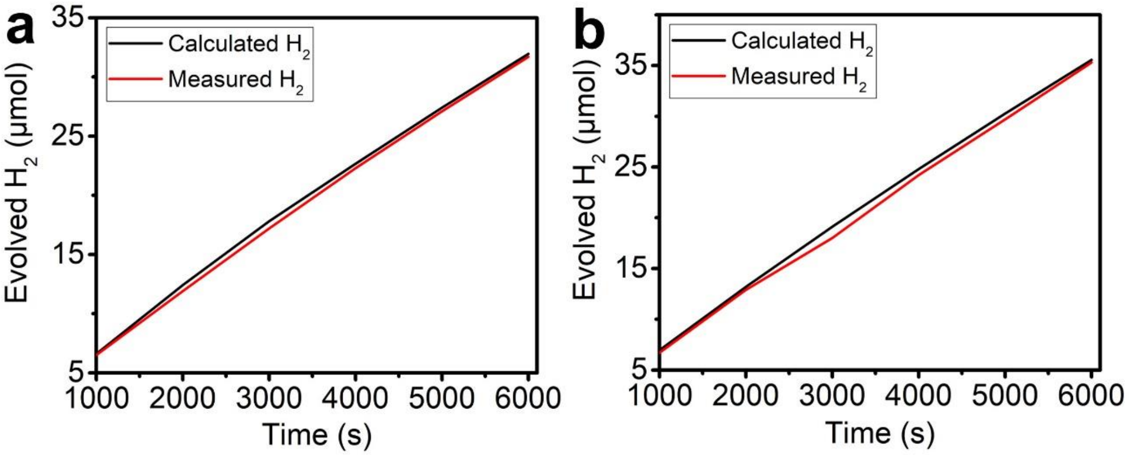

Supplementary Fig. 15 The evolved H_2 measured with GC (red) and theoretical volume (black) versus time obtained in **a** 0.5 M H_2SO_4 and **b** 1 M KOH condition. Faraday efficiencies higher than 97 % (calculate on the data of 6000 s).

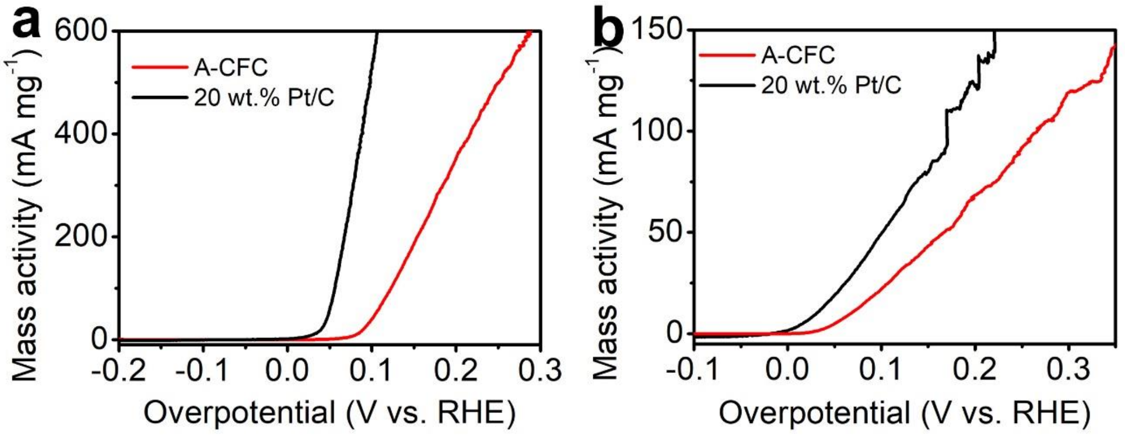

Supplementary Fig. 16 Mass activities of the catalysts obtained in **a** 0.5 M H_2SO_4 and **b** 1 M KOH.

Supplementary Tables

Supplementary Table 1 Comparison of the HER performances between A-CFC with recently reported benchmarked electrocatalysts obtained in 0.5 M H_2SO_4 condition.

**Electrocatalysts**	**η (mV) at $j_{10\ mA\ cm^{-2}}$**	**Tafel slope (mV dec^{-1})**	**j_0 (mA cm^{-2})**	**Ref.**
A-CFC	78	37	0.1	This work
c-CFC	457	103.7	0.00036	
3D graphene networks	107	64	0.0416	*Angew. Chem. Int. Ed.* **2018**, *57*, 192–197.
NSC/MPA-5	331	99	0.0048	*Nano Energy* **2017**, *32*, 336–346.
ONPPGC/OCC	470	200		*Energy Environ. Sci.* **2016**, *9*, 1210–1214.
BCN-1	298	100	-	*Energy Environ. Sci.* **2016**, *9*, 95–101.
Defect Graphene	150	55	-	*Adv. Mater.* **2016**, *28*, 9532–9538.
NSP750	240	90	-	*Adv. Mater.* **2016**, *28*, 10644–10651.
MPSA/GO-1000	163	89	-	*Angew. Chem. Int. Ed.* **2016**, *55*, 2230–2234.
g-C_3N_4 nanoribbon-G	207	54	0.0398	*Angew. Chem. Int. Ed.* **2014**, *53*, 13934–13939.
C_3N_4@NG hybrid	240	51.5	0.00035	*Nat. Commun.* **2014**, *5*, 3783.
Pt NWs/SL-$Ni(OH)_2$	95	--	--	*Nat. Common.* **6**, 6430 (2015)
CoP/NCNHP	140	53	--	*J. Am. Chem. Soc.* **140**, 2610–2618 (2018)
Cu_3P@NPPC-650	89	76	--	*Adv. Mater.* **30**, 1703711 (2018)
CoPS	145	48	0.0056	*Nat. Mater.* **14**, 1245–1251 (2015)
MoC_x nano-octahedra	142	53	--	*Nat. Common.* **6**, 6512 (2015)
CoN_x/C	133	57	0.096	*Nat. Common.* **6**, 7992 (2015)
$MoS_{2(1-x)}Se_{2x}$	69	42		*Nat. Common.* **7**, 12765 (2016)

$WO_{2.9}$	70	50	0.4	*Nat. Common.* **6**, 8064 (2015)
$Ni_{4.5}Fe_{4.5}S_8$	280	29		*Nat. Common.* **7**, 12269 (2016)
CoS|P/CNT	64	100		*Nat. Common.* **7**, 10771 (2016)
Co-NG	147	82		*Nat. Common.* **6**, 8668 (2015)

Supplementary Table 2 Comparison of the HER activity of the A-CFC in 1 M PBS with other reported metal-free and transition metal-based electrocatalysts.

**Electrocatalysts**	***j*** **(mA cm^{-2})**	***η*** **(mV) @** ***j***	**References**
A-CFC	2	230.8	This work
	10	436	
PPANI750	10	~580	*J. Am. Chem. Soc.* **137**, 15070-15073 (2015).
Co_3S_4	10	~480	*J. Am. Chem. Soc.* **138**, 1359-1365 (2016)
H_2-CoCat film	2	385	*Nat. Mater*. **11**, 802-807 (2012).
Co-S/FTO	10	~720	*J. Am. Chem. Soc.* **135**, 17699-17702 (2013)
Co-NRCNTs	10	~ 540	*Angew. Chem. Int. Ed.* **53**, 4372-4376 (2014)

Supplementary Table 3 Summary of EIS fitting parameters for A-CFC and c-CFC.

Catalysts	R_s (Ω)	R_{ct} (Ω)	n_1	Q_{ct}	R_h (Ω)	n_2	Q_h
A-CFC	3.14	0.69	0.89	8.7×10^{-4}	245.7	0.95	8.1×10^{-4}
c-CFC	5.39	0.81	1	9.8×10^{-5}	453.7	0.85	1.1×10^{-3}

Supplementary Table 4 Comparison of HER activities of A-CFC with c-CFC at an overpotential of 300 mV in 1.0 M KOH.

**Catalysts**	**Specific Activity (mA cm^{-2})**	
	$^{a}j_{geo}$	$^{b}j_{ECSA}$
A-CFC	78.9	0.06
c-CFC	2.86	0.02

[a]Values calculated by geometric area; [b]Values calculated by ECSA.

Supplementary Table 5 Comparison of the mass activity of A-CFC with state-of-the-art metal-free HER electrocatalysts in acidic/alkaline aqueous media.

**Catalysts**	**Mass activities**	**Electrolytes**	**References**
A-CFC	186.1 mA mg^{-1} at 150 mV<br>353.6 mA mg^{-1} at 200 mV	0.5 M H_2SO_4	This work
	43.2 mA mg^{-1} at 150 mV<br>119 mA mg^{-1} at 300 mV	1 M KOH	
C_3N_4@NG	100 mA mg^{-1} at 240 mV	0.5 M H_2SO_4	*Nat. Commun.* **4**, 3783 (2014)
NS-doped hierarchical nanoporous graphene	28.6 mA mg^{-1} at 230 mV	0.5 M H_2SO_4	*Angew. Chem. Int. Ed.* **57**, 13302–13307 (2018)
B/P/S-Doped g-C3N4	35.7 mA mg^{-1} at 186 mV	0.5 M H_2SO_4	*ACS Nano* **11**, 6004−6014 (2017)
g-CN@G MMs	70.4 mA mg^{-1} at 219 mV	0.5 M H_2SO_4	*Adv. Funct. Mater.* **27**, 1606352 (2017)
Defective graphene	35.1 mA mg^{-1} at 320 mV	1 M KOH	*Adv. Mater.* **28**, 9532–9538 (2016)
N, P and O tri-doped porous graphite carbon@oxidized carbon cloth	100 mA mg^{-1} at 450 mV	1 M KOH	*Energy Environ. Sci.* **9**, 1210–1214 (2016)

Supplementary Note 1

Computational Methods. We performed the density functional theory (DFT) calculations based on the CASTEP code[1]. The PBE functional is chosen and related plane wave basis set cut-off energy is set at the 750 eV based on a denser Monkhost-Pack k-point mesh of 4×4×2. The ensemble DFT (EDFT) has been used for improving the electronic minimization to overcome the spurious spin-charge perturbation seen in density-mixing scheme[2]. The convergence tolerance of total energy calculation is determined at 5.0 x 10^{-7} eV/atom with ionic force minimization level of 0.001 eV/Å by Hellmann-Feynman theorem. The C, N, O, and H norm-conserving pseudopotentials are generated using the OPIUM code in the Kleinman-Bylander projector form[3] and a scalar relativistic averaging scheme[4] are selected to treat the spin-orbital coupling effect. We chose the (2*s*, 2*p*) and (1s) states as the valence states of C, N, O, and H atoms respectively. The RRKJ method is chosen for the optimization of the pseudopotentials[5], which has been tested in recent published works[6].

The additional energy barrier for water dissociation as well as the poor water binding energies are still great challenges. Rather than the simple calculations of adsorption of hydrogen, which has considered the binding behaviors of water and OH group. The HER in alkaline usually include three types of reactions as Volmer reaction, Heyrovsky reaction and the Tafel reactions that are described as follows[7,8]:

$$H_2O + \ e^- + Catal \ \rightarrow OH^- + Caltal - \ H^* + \ e^- \qquad (Volmer\ \ step)$$

$$Catal - \ H^* + \ H_2O + \ e^- \ \rightarrow \ H_2 + \ OH^- \qquad (Heyrovsky\ \ step)$$

or

$$2\text{Catal} - \ H^* \ \leftrightarrow \ H_2 + 2Catal \qquad\qquad (Tafel\ \ step)$$

Catal denotes to the surface of catalyst and Catal-H* depicts hydrogen intermediates adsorbed on the surface. The onset potential of HER is determined by a Volmer step. When the Volmer, Heyrovsky, or Tafel step is the rate-determining step, respectively, different Tafel slopes can be derived from Butler−Volmer kinetics[9,10]. Therefore, by compare the free energy diagram of these reactions on different surfaces, the preferred catalyst with facile barriers can be determined. In this work, we have calculated the kinetic energy barrier of the prior Volmer step (ΔG(H_2O)) and the concomitant combination of adsorbed H into molecular hydrogen (ΔG(H), Heyrovsky or Tafel step). The reduced ΔG(H_2O and ΔG(OH) values on catalysts can suggest that the kinetics of the initial water dissociation step and the concomitant desorption of the formed OH can be effectively promoted, which can turn a sluggish Volmer–Tafel step to a fast Tafel step reaction. The optimized geometries of our models along the reaction coordinates are following stringent mass transfer with the charge balance in HER of the alkaline condition, which should be distinguished with the simple acidic condition. Moreover, the calculation of HER in alkaline condition can facilitate the understanding of the bi-functional catalysts that can apply in the pH-universal environment.

Therefore, the simulation of the alkaline environment with the involvement of water and OH in the HER is very critical to precisely reveal the reactivity of catalyst in the reactions.

For all the DFT calculation on Gibbs free energies in this work, all the changes of entropy and zero-point energy have been taken into account. The energy profile is plotted based on:

$$\Delta\text{G} = \ \Delta\text{E} - \text{T}\Delta\text{S} + \ \Delta\text{ZPE} \qquad (1)$$

where ΔE is the reaction energy, ΔS is the entropy change, and ΔZPE is the zero-point energy difference. From the energy diagram, we can clearly observe the binding preference, overbinding and desorption difference in different surfaces. The related formation energies have been computed based on the equation developed by Zunger *et al.* within the CASTEP package[11]. The overall supercell was established and remained fixed for all lattice parameters based on the ground state relaxed primitive cell to reduce the effect of enthalpy changes resulting from cell variations. All the geometry optimizations have been operated based on the Broyden-Fletcher-Goldfarb-Shannon (BFGS) algorithm. For those non-neutral charge calculations, a Coulomb potential correction will be required to offset the impacts from the image charge of the crystal lattice. The PBE functional was chosen for PBE+U calculations with kinetic cutoff energy of 750 eV, with the valence electron states expressed in a plane-wave basis set. The ensemble DFT (EDFT) method of Marzari *et al.* is used for convergence on the transition metal contained compounds[12]. The RRKJ method is chosen for the optimization of the pseudopotentials[13].

Supplementary Note 2:

The TOF is calculated according to the following equation:

$$\text{TOF} = \frac{Total\ \ number\ \ of\ \ H_2\ \ molecules\ \ per\ \ second}{Total\ \ number\ \ of\ \ active\ \ sites\ \ per\ \ unit\ \ area} = \frac{j}{2qN} \qquad (2)$$

where j is the current density, q is the elementary charge as 1.6×10^{-19}, N is the active site density, and 2 means the electron transfer number as one hydrogen molecule generation. The active sites per unit area can be estimated from the electrochemical surface area (ECSA = 235 cm^2, please see Methods Section in the manuscript for details).

According to our experimental and theoretical results, the active sites have been demonstrated to be related the amide groups. The upper limit number of HER active sites in A-CFC was calculated based on the hypothesis that N atoms on the A-CFC surface formed the active centers accessible to the electrolyte. The percentage of N was obtained from the EDS results (~1.44 wt.%). The average mass of the A-CFC electrode is 0.125 mg, and the geometric surface area of the electrode is 0.1 cm^2. The active sites density (N) can be calculated according to the following equation:

$$\text{N} = \frac{n \times N_A}{A} \qquad (3)$$

where n is the mole of the active centers, N_A is the Avogadro constant, and A corresponds to the geometric surface area of the electrode.

Therefore,

$$\text{Active sites density (N)} = \frac{n \times N_A}{A} = \frac{\frac{(0.125\ \ \text{mg} \times 1.44\ \ \text{wt.\%})/1000}{14\ \ g\ \ mol^{-1}} \times (6.022 \times 10^{23})}{0.1\ \ cm^2}$$

$$= \ 7.74\ \ \times 10^{17}\ \ sites\ \ cm^{-2} \qquad (4)$$

The TOF of A-CFC at different overpotentials is calculated as follows:

In 0.5 M H_2SO_4,

At 100 mV, $\text{TOF} = \frac{j}{2qN} = \frac{j}{2\times1.6\times10^{-19}\times7.74\times10^{17}\times235} = \frac{50.1}{58.2} = 0.86\ s^{-1}$ (5)

In 1 M KOH,

At 100 mV, $\text{TOF} = \frac{24.11}{58.2} = 0.41\ s^{-1}$ (6)

Similarly, the TOF of c-CFC is 0.08 s^{-1} and 0.14 s^{-1} at the overpotential of 100 mV in 0.5 M H_2SO_4 and 1 M KOH, respectively.

Supplementary references:

[1] Clark, S. J. *et al.* First principles methods using CASTEP. *zkri* **220**, 567 (2005).

[2] Marzari, N., Vanderbilt, D. & Payne, M. C. Ensemble Density-Functional Theory for *Ab Initio* Molecular Dynamics of Metals and Finite-Temperature Insulators. *Phys. Rev. Lett.* **79**, 1337-1340 (1997).

[3] Kleinman, L. & Bylander, D. M. Efficacious Form for Model Pseudopotentials. *Phys. Rev. Lett.* **48**, 1425-1428 (1982).

[4] Grinberg, I., Ramer, N. J. & Rappe, A. M. Transferable relativistic Dirac-Slater pseudopotentials. *Phys. Rev. B* **62**, 2311-2314 (2000).

[5] Rappe, A. M., Rabe, K. M., Kaxiras, E. & Joannopoulos, J. D. Optimized pseudopotentials. *Phys. Rev. B* **41**, 1227-1230 (1990).

[6] Xue, Y. *et al.* Anchoring zero valence single atoms of nickel and iron on graphdiyne for hydrogen evolution. *Nat Commun* **9**, 1460 (2018).

[7] Subbaraman, R. *et al.* Enhancing hydrogen evolution activity in water splitting by tailoring Li^+-$Ni(OH)_2$-Pt interfaces. *Science* **334**, 1256–1260 (2011).

[8] Danilovic, N. *et al.* Enhancing the alkaline hydrogen evolution reaction activity through the bifunctionality of $Ni(OH)_2$/metal catalysts. *Angew. Chem. Int. Ed.* **124**, 12495–12498 (2012).

[9] Conway, B. E. & Tilak, B. V. Interfacial processes involving electrocatalytic evolution and oxidation of H_2, and the role of chemisorbed H. *Electrochim. Acta* **47**, 3571−3594 (2002).

[10] Mahmood, N. *et al.* Electrocatalysts for hydrogen evolution in alkaline electrolytes: mechanisms, challenges, and prospective solutions. *Adv. Sci.* **5**, 1700464 (2017).

[11] Lany, S. & Zunger, A. Assessment of correction methods for the band-gap problem and for finite-size effects in supercell defect calculations: Case studies for ZnO and GaAs. *Phy. Rev. B*, **78**, 235104 (2008).

[12] Marzari, N., Vanderbilt, D. & Payne, M. C. Ensemble density-functional theory for *Ab Initio* molecular dynamics of metals and finite-temperature insulators. *Phys. Rev. Lett.* **79**, 1337 (1997).

[13] Rappe, A. M., Rabe, K. M., Kaxiras, E. & Joannopoulos, J. Erratum: optimized pseudopotentials [Phys. Rev. B 41, 1227 (1990)]. *Phys. Rev. B* **44**, 13175 (1991).
